# The complete mitochondrial genome of sleek unicornfish, *Naso hexacanthus* (Acanthuridae, Perciformes)

**DOI:** 10.1080/23802359.2022.2160666

**Published:** 2023-02-20

**Authors:** Hae-young Choi, Sung Kim, Hee-chan Choi, Seok-hyun Youn

**Affiliations:** a Fisheries Resources and Environment Division, South Sea Fisheries Research Institute, Yeosu, Korea; b Marine Ecosystem Research Center, Korea Institute of Ocean Science & Technology, Busan, Korea; c Fisheries Resources and Environment Division, East Sea Fisheries Research Institute, Gangwon, Korea

**Keywords:** Acanthuridae, complete mitogenome, *Naso hexacanthus*

## Abstract

The complete mitochondrial DNA sequence of sleek unicornfish, *Naso hexacanthus* was first determined in this study. The complete mitogenome is 16,611 bp in length composed of 13 protein-coding genes, 2 ribosomal RNAs, 22 transfer RNAs, and a control region. The nucleotides consist of 33.8% A, 20.6% C, 25.0% G, 20.6% T. The gene order and direction are identical to those of *N. lopezi* and the species of Acanthuridae. The result would be useful to investigate genetic relationships among the species of *Naso*.

The genus *Naso* (Perciformes: Acanthuridae) including 20 species (Guala 2019) are coral reef fishes, widely distributed in subtropical and tropical areas (Fricke et al. 2021), ecologically and morphologically diverse (Randall 2002). To understand the relationship among the species of *Naso,* partial mitochondrial DNA (mtDNA) sequences have been used in various studies (Klanten et al. 2004; Ho et al. 2011). Genetic population structure of three species, *N. brevirostris*, *N. unicornis* and *N. vlamingii* are compared based on control regions of the mtDNA (Horne et al. 2008). However, phylogeographical characteristics between *N. hexacanthus* and *N. caesius* are not distinguished by COI sequences (Horne and van Herwerden 2013). Complete mitogenome information is useful to search for appropriate markers. But the complete mitogenome of *Naso* species was analyzed for only one species, *N. lopezi*. Here, we report the first complete mitogenome of *N. hexacanthus*, which could be the basis for further molecular study.

A specimen of larval *N. hexacanthus* used to generate the mitogenome, was collected from the East China Sea (32.5000°N, 127.0873°E) on 27 August 2021 and identified based on DNA barcodes and morphological characteristics (Choi et al. 2022). Genomic DNA of the specimen was extracted from its eye followed by the protocol of a DNeasy Blood & Tissue Kit (Qiagen, Hilden, Germany). The genomic DNA and specimen were stored in the National Institute of Fisheries Science (http://www.nifs.go.kr/, Seok-hyun Youn, younsh@korea.kr) under voucher number 2108ECS31513L. The genomic DNA was used to make a sequencing library for NovaSeq 6000 (Illumina, San Diego, CA) using a Nextera DNA Flex Library Prep kit according to the protocol. The massive nucleotide sequences were mapped to a reference sequence, *Naso lopezi* (NC_009853.1) using each Bowtie2 (Langmead and Salzberg 2012) and Geneious mapper in Geneious R11 (Biomatters, Auckland, New Zealand) (Kearse et al. 2012). A consensus sequence constructed from the mapping (mean coverage ± standard deviation, 1347.2 ± 321.4) was annotated in each MitoFish (Iwasaki et al. 2013) and Geneious R11.

The complete mitochondrial mitogenome of *Naso hexacanthus* (GenBank accession number: OM494539) was 16,611 bp in length and consisted of 13 protein-coding genes (PCGs), 2 ribosomal RNAs (rRNAs), 22 transfer RNAs (tRNAs), and a control region. The nucleotide compositions were 33.8% A, 20.6% C, 25.0% G, and 20.6% T. The start codon of 12 protein-coding genes was ATG, except GTG for *cox1*. The stop codons were TAA for 5 PCGs (*atp8*, *cox1*, *nd1*, *nd4l* and *nd5*), TAG for one PCG (*nd6*), and incomplete stop codons, TA (*atp6*, *cox3* and *nd2*) and T (*cox2*, *cytb*, *nd3*, *nd4*). The positions and directions of the genes were identical to those of other Acanthuridae (Yamanoue et al. 2007; Huang et al. 2017; Ludt et al. 2020).

To investigate the relationship between the *N. hexacanthus* and species of Acanthuridae, a maximum likelihood tree was constructed based on GTR + G + I model (Nei and Kumar 2000) in MEGA-X (Tamura et al. 2013). Concatenated sequences comprised of 13 PCGs and 2 rRNAs from the *N. hexacanthus*, species of Acanthuridae, and outgroups were used for this analysis. In the phylogenetic tree, *N. hexacanthus* was distinguished from *N. lopezi* (Figure 1).

**Figure 1.**
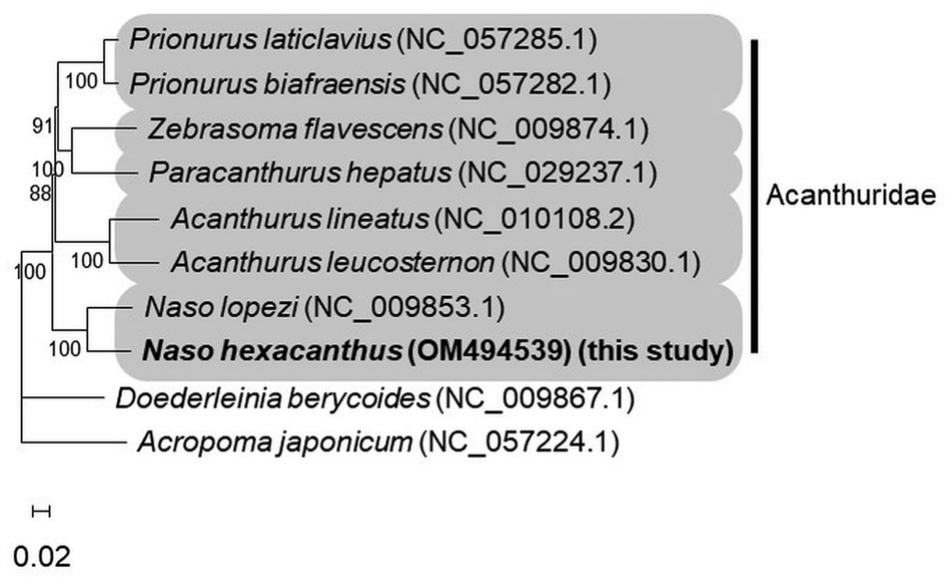
Maximum likelihood tree constructed using concatenated sequences of 13 PCGs and 2 rRNAs from the *Naso hexacanthus*, species of Acanthuridae, and outgroups. Numbers are bootstrap values (1000 replicates) greater than 50%.

Our complete mitochondrial DNA sequence of *Naso hexacanthus*, obtained by deep sequencing would be useful to understand the relationships of *Naso* species.

## Data Availability

The genome sequence data that support the findings of this study are openly available in GenBank of NCBI at [https://www.ncbi.nlm.nih.gov] (https://www.ncbi.nlm.nih.gov/) under the accession no. OM494539. The associated BioProject, SRA, and Bio-Sample numbers are PRJNA841571, SRR19355784, and SAMN28597496 respectively.
